# Preventing mental health problems in offspring by targeting dietary intake of pregnant women

**DOI:** 10.1186/s12916-014-0208-0

**Published:** 2014-11-14

**Authors:** Adrienne O’Neil, Catherine Itsiopoulos, Helen Skouteris, Rachelle S Opie, Skye McPhie, Briony Hill, Felice N Jacka

**Affiliations:** IMPACT Strategic Research Centre, School of Medicine, Deakin University, PO Box 281, Geelong, Victoria 3220 Australia; School of Public Health and Preventive Medicine, 89 Commercial Road; Alfred Hospital, Prahran, Victoria 3004 Australia; Department of Dietetics and Human Nutrition, Faculty of Health Sciences, Latrobe University, Melbourne, Victoria 3086 Australia; School of Psychology, Deakin University, 221 Burwood Highway, Burwood, Victoria 3125 Australia; Centre for Adolescent Health, Murdoch Children’s Research Centre, Royal Children’s Hospital, 50 Flemington Rd, Parkville, Victoria 3052 Australia; Department of Psychiatry, University of Melbourne, Level 1 North, Main Block, Royal Melbourne Hospital, Melbourne, Victoria 3050 Australia; Black Dog Institute, Prince of Wales Hospital, Hospital Rd, Randwick, NSW 2013 Australia

**Keywords:** Antenatal, Anxiety, Depression, Diet quality, Early life programming, Maternal diet, Offspring, Perinatal, Pregnancy, Prevention

## Abstract

**Background:**

The concept of ‘early life programming’ considers the importance of very early environmental exposures throughout the gestational period on the subsequent health outcomes of offspring. The role of maternal dietary intake, specifically, has been highlighted after recent studies have shown maternal diet quality to predict mental health problems in offspring. Even in the pre-conception period, maternal nutrition can have permanent and sustained phenotypic consequences for offspring.

**Discussion:**

Here, we consider these findings in the context of the primary prevention of mental disorders and argue that interventions that target maternal diet could be of significant value.

**Summary:**

It is clear that, in order to reduce the burden of mental health issues across the lifespan, urgent action is required, particularly in the field of prevention. We thus call for the application and evaluation of targeted, primary prevention strategies that focus on dietary intake with the view to improve mental health outcomes of mothers and offspring during the postnatal period and beyond.

## Background

Mental disorders are significant contributors to the global burden of disease. Major depression accounts for the second highest burden of disability worldwide [1]. While certain populations are at greater risk of experiencing mental health problems such as depression, these conditions can affect individuals across the entire lifespan, including childhood [2]. In fact, half of mental illnesses first manifest prior to 14 years of age [3] and these tend to be enduring [4]. Current estimates suggest that the lifetime prevalence of mental disorders in the United States is 46.3% in 13 to 18 year olds [5]. As childhood disorders can be a risk factor for adolescent suicide [6], as well as for a range of long-term deleterious social, criminal, and economic outcomes in adulthood [7], there is a clear imperative to employ primary prevention strategies that target depression early in the lifespan.

Traditionally, efforts to address the burden of depression have focused on treatment rather than prevention. Preventive interventions for mental disorders in children and adolescents have often involved the use of established psycho-therapeutic approaches, delivered in community, school, and online settings [8], but have not employed approaches to primary prevention that focus on lifestyle modification. In contrast, such strategies to reduce the incidence of high prevalence, chronic somatic conditions as, for example, cardiovascular disease and some cancers, have been effective over past decades [9,10]; dietary improvement, in particular, has been demonstrated to be an effective approach [11].

### The role of habitual diet and mental disorders

Recent evidence from observational studies suggests that habitual dietary intake can play a fundamental role in the risk for and progression of common mental disorders [12,13]. Thus, it seems plausible that a proportion of the burden of mental illnesses is potentially preventable by targeting dietary behaviors. Additionally, the importance of early environmental exposures throughout and prior to the gestational period (termed ‘early life programming’ [14]) and in early childhood has recently emerged. In large and well characterized cohort studies, maternal diet during pregnancy is related to the risk for behavioral problems in offspring [15,16]. Further studies have elucidated the potential importance of maternal dietary intake in humans by demonstrating that early maternal nutrition (stage of gestation at the time of sampling = 8.6 ± 4.0 weeks) causes persistent and systemic epigenetic changes in phenotype of offspring at 2 to 8 months of age (Mean: 3.6 months, Standard Deviation ± 0.9) [17]. Moreover, recent animal studies have indicated that the father’s preconception diet may be of equal importance to the health of offspring; in the sperm of male mice, folate deficiency alters the methylation of genes implicated in the development of diseases including autism and schizophrenia [18].

### Habitual diet is linked to mental health problems in children and adolescents

Over the past five years, data have emerged to support the relationship between the mental health of children and their ‘dietary patterns’, often classified by factor, principal component, or latent class analysis of food frequency data. A number of studies in this area have demonstrated that ‘unhealthy’ childhood dietary patterns are linked to poorer mental health, including internalizing and externalizing behaviors. The former term encompasses symptoms of depression, anxiety, and sadness, while the latter captures anger, aggression, and hyperactivity; both are commonly used as a marker of mental health problems in children [19]. We recently performed a systematic review of the (albeit limited) evidence base [20] and demonstrated a significant, cross-sectional relationship between ‘unhealthy’ dietary patterns and poorer (internalizing) mental health in children and adolescents. A consistent trend was observed for the relationship between good diet quality and better mental health, and some evidence for poor diet quality being related to worse mental health. Two of the three major cohort studies investigating these prospective associations yielded positive findings for the link between diet quality/variety and internalizing disorders; one in children [21] and the other in adolescent populations [22]. Notwithstanding the paucity of longitudinal data in this area of research, these findings suggest that childhood nutritional exposures have the potential to contribute to the development of mental health problems in childhood and adolescence. This evidence base has prompted recent investigation into the relationship of diet quality to mental health in children even earlier in the lifespan, when brain development is most rapid – specifically, during the antenatal period.

### Maternal diet is a predictor of poor mental health of offspring

During pregnancy, and particularly early pregnancy, exposure of the developing fetus to an unfavorable environment predisposes the unborn child to numerous chronic diseases [23]. There is an established link between maternal diet and neurological, immunological, and central nervous system development of offspring, all of which can play a crucial role in the subsequent development of mental disorders. Data from animal models support this idea, demonstrating a link between perinatal essential fatty acid deficiencies and poorer biological development (e.g., reduced brain plasticity) of offspring related to mood disorders [24]. Animal studies have further demonstrated the impact of poor maternal nutrition (e.g., high saturated fats and high added sugar) on the activation of the sympathetic nervous system and hyperactivity behaviors, which may have implications for behaviors related to adult mood disorders [25]. Recently, these findings have been replicated in human studies.

A recent investigation has revealed the predictive role of dietary intake during pregnancy on subsequent mental health of offspring [15]. Data were derived from the large prospective Norwegian Mother and Child Cohort Study, which recruited pregnant women between 1999 and 2008 and monitored mothers during pregnancy and again when children were 6 months, 18 months, 3 years, and 5 years old. An unhealthy dietary pattern during pregnancy was characterized by the consumption of processed meat products, refined cereals, sweet drinks, and salty snacks. An unhealthy dietary pattern in children was characterized by high fat/sugar foods such as chips, cakes, chocolate, sweets, soft drinks, ice cream, and pizza. A ‘wholesome’ dietary pattern during pregnancy was characterized by vegetables, fruit, high-fiber cereals, and vegetable oils. A ‘wholesome’ dietary pattern in children was characterized by an increased consumption of foods including white and oily fish, vegetables, fruit, and eggs. Using data from 23,020 eligible women and their children, higher scores on an unhealthy dietary pattern during pregnancy were shown to predict increased externalizing problems among children, independent of other potential confounding factors and childhood diet. A similar trend was observed when the diet of the children in the postnatal period was explored; high levels of unhealthy diet predicted higher levels of both internalizing and externalizing problems in children. Moreover, children with lower scores on the wholesome dietary pattern also had higher levels of internalizing and externalizing problems. When the authors monitored the trajectory of internalizing behaviors for children with high or low unhealthy dietary intakes, a convergence in these trajectories was observed over the 5-year follow-up period, suggesting that the contribution of unhealthy foods to internalizing behaviors is most potent in early life (but may diminish over time). However, the impact of nutritional exposures on externalizing behaviors remained over this period. In contrast, the difference in internalizing and externalizing scores over time for those high and low on nutrient-dense foods tended to diverge over the measured period, suggesting that the impact of inadequate nutrition in early childhood on mental health vulnerability may be more profound the longer the period of exposure. A further crucial finding of this study relates to the magnitude of the relationships between diet and mental health in children. In some cases, the size of the standardized coefficients for the dietary exposures were comparable to that of maternal depression, which is an established risk factor for poor mental health in children [15]. This finding was replicated more recently in the Gen-R population-based, cohort study in the Netherlands [16]. In this study of more than 3,000 children, mothers’ diets during pregnancy were assessed and three main dietary patterns identified: a Mediterranean (healthy), Traditionally Dutch (characterized by higher intakes of fresh and processed meats, potatoes and margarines), and a Confectionary dietary pattern. Following adjustment for a number of potentially explanatory factors, including parental education and income, psychopathology, and child consumption of snacks and sugars, they reported that lower scores on the Mediterranean dietary pattern and higher scores on the Traditional Dutch pattern during pregnancy were both independently associated with higher levels of externalizing in offspring.

The idea that early maternal nutrition – and perhaps periconception nutrition – is a potent predictor of the health of offspring is further supported by other studies. Data from the Southampton Women’s survey have shown the importance of specific macronutrients (carbohydrates during the first trimester and protein during the third trimester) on the size of offspring at birth [26]. A seminal study by Dominguez-Salas et al. [17] reported on women who conceived throughout different seasonal periods in Gambia, during which times food consumption is subject to pronounced variations in macro and micronutrient intake that have long been known to affect fetal growth and development. The authors performed a prospective study designed to track the influence of mothers’ periconceptional dietary intakes and plasma concentrations of key methyl-donor pathway substrates; the first human study to do so. Measured at the time of conception, maternal blood biomarkers of substrates and cofactors (required for methyl-donor pathways) were significant predictors of epigenetically-modifiable alleles in DNA extracted from offspring in the postnatal period (using lymphocytes and hair follicles). The authors concluded that maternal nutritional status during early pregnancy “*causes persistent and systemic epigenetic changes at human metastable epialleles*” [17]. Further studies are attempting to distinguish the extent to which the effects of diet are driven by adiposity or energy balance. However, this study was able to eliminate many putative confounders and supports findings from earlier animal models.

Not only do these studies highlight the importance of maternal diet in offspring development, but they also point to the potential of dietary manipulation in the periconception and perinatal period as a key strategy for improving health outcomes in children. Early pregnancy or family planning periods may provide ‘teachable moments’ for improving dietary behaviors to reduce the risk of disorder onset in offspring. This is largely attributed to frequent contact with family planning or health care providers and higher levels of motivation during this time [27]. It is with this in mind that we argue for maternal dietary modification as a useful starting point in the primary prevention of mental disorders of offspring. Such an approach has the potential to act as a ‘double hit’ in also preventing mental health issues in mothers.

## Discussion

### Primary prevention of mental health problems in offspring requires effective prevention and treatment of symptoms in mothers

We first need to acknowledge that maternal depression exacerbates a child’s risk of developing a mental disorder later in life. The presence of antenatal depression equates to a five-fold increase in offspring developing the condition by the time they are a teenager [2]. The reasons for this are multifaceted and likely have an epigenetic and behavioral basis [28]. For example, the evidence suggests that maternal depression is linked to being non-responsive to infant needs [29] and to compromised postnatal behaviors, including inadequate or absence of breastfeeding [30]. Breastfeeding is considered optimal nutrition in the first six months of life [31] for the infant due to its ability to support and develop a baby’s immune system [32], as well as prevention of later obesity, metabolic syndrome, cardiovascular disease, and behavioral and emotional problems in the child [14].

These findings suggest that a crucial component of primary prevention of mental disorders in children involves the adequate and timely prevention and treatment of depression in mothers. It is known that women are particularly susceptible to developing depression during their child-bearing years and this risk is further exacerbated for women during pregnancy, particularly considering only a small proportion (6%) seek help for depression [33]. Given the negative health outcomes of maternal depression for both mothers and children, there have been calls for mandatory formal depression screening to be included in antenatal clinics to allow those with depressive symptoms to be referred to early intervention initiatives, be monitored during their pregnancy, and referred to treatment options as necessary [34]. For example, in Australia, the Government initiated the National Perinatal Depression Plan in 2008 to address ante- and postnatal depression. It recommended routine screening and treatment of ante- and postnatal depression by upskilling healthcare professionals [35]. There remains, however, variability in how this is carried out in regards to gestational stage, scoring, and response/s to potential depression [36]. Currently, standard treatments for maternal depression include psychotherapy and/or pharmacotherapy; however, such treatments are only effective for less than half of the population [37] and may be contraindicated during pregnancy. For example, while pharmacotherapy targets the physiological genesis of depression encompassing reduced dopamine, serotonin, and norepinephrine levels, and alterations in hormones and neuro-membranes, there have been safety concerns surrounding the use of some therapeutic agents in pregnancy [38]. The appeal of non-pharmacological approaches, such as dietary modification, in the prevention or treatment of depression in pregnant women relates to the limited side effects and treatment costs of more conventional approaches [39]. Hence, there is burgeoning interest in the association between maternal dietary quality and mental health of mothers. Overall trends suggest that poor quality maternal diet is associated with antenatal depressive [40-42], anxiety [43], and stress symptoms [41,42] as well as postpartum depressive symptoms [40]. Importantly, when the association with ‘traditional’ diets (e.g., vegetables, lean red meat, poultry) was reviewed, healthy diets were inversely associated with antenatal and postpartum depressive symptoms [40] and antenatal anxiety [43]. This alludes to the promise of dietary modification in the antenatal period to improve both maternal and childhood mental health.

### Nutritional interventions for pregnant women: a single-nutrient versus whole-diet approach?

Nutritional interventions are often endorsed for depression in pregnant women, given their potential to alter aforementioned pathways via consumption of specific nutrients (e.g., docosahexaenoic acid, B vitamins, folate) that promote neurotransmitter synthesis and absorption [39]. While there is evidence from experimental studies that micronutrient supplementation during pregnancy can lead to reductions in maternal depressive symptoms as well as improving birth outcomes [39], there have been criticisms of employing a purely supplementation approach to treat diet-related health problems [44]. For example, while large epidemiological studies have consistently shown that higher intakes of omega-3 fatty acids from fish and seafood in the diet are associated with reduced risk of depressive symptoms in the postnatal period and improved developmental outcomes in the children [45], and that poor omega-3 status is associated with increased risk [46], supplementation trials with omega-3 fish oils have not shown protective effects, indicating that there may be other important nutrients in fish and seafood that are protective for maternal depression [44]. Thus, ‘whole-diet’ interventions for pregnant women, as distinct from supplementation interventions, have been advocated by some. However, most trials conducted in this area have focused on obesity prevention with only several specifically focused on mental health outcomes [47]. While these data provide promising evidence for the potential for healthy lifestyle interventions to reduce depressive symptomatology during pregnancy, they fail to provide insight as to whether dietary intervention alone can effectively prevent depressive symptomatology in this population and, importantly, whether these effects translate to benefits to offspring.

### ‘Whole-diet’ approach may be effective for the primary prevention of mental disorders

Preliminary evidence suggests that the protective effects of diet can extend to depression risk. Adherence to the Mediterranean-style diet, for example, has protective effects on brain diseases, including depression [48]. Embedded within the PREDIMED trial, Sánchez-Villegas et al. analyzed depression incidence in a sample of 3,923 participants at risk of cardiovascular disease after they were exposed to a dietary intervention over 3 years [49]. While the authors found the strongest reduction of risk in a sub-group of participants with type 2 diabetes, they estimated that exposure to the Mediterranean diet supplemented with nuts accounted for a reduction in depression risk of approximately 25%, compared with a low fat diet. While we recognize that exceptionally large sample sizes are required to demonstrate primary prevention of depression for any exposure, these data support the hypothesis that depression may be somewhat preventable through ‘whole-diet’ approaches. A review of randomized trials conducted by our team has highlighted the benefits of this approach for improving mental health in various medical populations [50]. Indeed, these findings may be generalizable to pregnant women, conferring benefit to the mental health of their children due to its beneficial components for brain health, such as B-group vitamins and long chain omega-3 fatty acids, rich in bioactive phytonutrients with antioxidant and anti-inflammatory potential.

### Is it feasible to apply this approach to pregnant populations?

From a feasibility perspective, there are pilot data to support the contention that diet is readily modifiable in pregnant women [51]. Indeed, a meta-analysis demonstrated that dietary interventions administered throughout pregnancy tend to be more effective than those targeting physical activity [52]. While only a few trials have studied the effects of dietary modification in pregnancy, the results generally indicate good dietary compliance [53], in some cases as high as 77.6% [54]. However, we also know that without intervention, women do not tend to deviate from their regular dietary patterns during the early stages of pregnancy, and may even experience deterioration in their diet quality over the duration of their pregnancy [55]. For example, there is a tendency for women to consume a ‘high-energy’ diet in later stages of pregnancy, when compared with pre-pregnancy [56]. While this may be due to a range of factors driven by appetite and hormonal changes, nausea, food aversions, or an inadequate knowledge of antenatal dietary recommendations, evidence of worsening habitual diet across the pregnancy suggests there is scope to improve diet quality in pregnant women. To date, relevant health care advice provided to pregnant women has been shown to be limited [57]; however, promisingly, there is historical evidence of women’s receptiveness to health behavior. Dietary interventions targeted at pregnant women may thus be feasible and acceptable in clinical settings, while public health messages targeted at this population may have a positive impact on dietary behaviors.

### Is it too soon to propose population-level nutritional interventions to women of child-bearing age?

While there are extensive data from animal models demonstrating the impact of nutritional exposure on parameters relating to mental health outcomes, including neurotransmitter systems [58], brain plasticity [24], and the stress response system and related behaviors in offspring [25], a detailed exposition of the proposed pathways is beyond the scope of this paper. However, based on the extensive observational data, including evidence of both the reduced risk for depression and internalizing and externalizing behaviors as well as preliminary evidence for the prevention of depression with a dietary improvement in sub-populations, alongside the extensive pre-clinical data showing a potent impact of nutrition on prevention of chronic disease parameters in offspring, we believe that the delivery of health education and nutritional and lifestyle interventions to women during the antenatal period (when mothers are highly motivated and responsive to diet and lifestyle changes) is likely to have utility in ameliorating risk of a broader range of mental health disorders and cognition-related developmental delay outcomes, as well as having benefits for a wide range of other health outcomes in children [59]. While such interventions are not without their challenges [59], the burden currently imposed by mental health disorders globally, and the limitations of existing strategies to address the incidence and prevalence of such disorders, makes the attempt to implement universal prevention strategies such as these an imperative.

## Summary

Diet is a key modifiable risk factor for the development of non-communicable diseases, yet its role in the onset of mental disorders has received much less attention until very recently. There is now consistent evidence providing support for diet quality as a risk factor for depression. Evidence highlighting the potency of maternal diet on the mental health of offspring is emerging and is likely driven by diet-induced phenotypic changes at periconception and/or perinatally. This suggests that there may be great potential to target dietary intake during these pivotal times of fetal development. This paper has focused on the role of the perinatal and the ‘true’ postnatal period in which dietary intake has been demonstrated to be so important and which provides an accessible inflection point for intervention with women. That said, however, the importance of good quality dietary intake during early childhood and adolescence should not be ignored in the context of ameliorating risk of onset of a mental disorder. It is clear that in order to reduce the burden of mental health issues across the lifespan, urgent action is required, particularly in the field of prevention. We thus call for the application and evaluation of targeted, primary prevention strategies that focus on dietary intake with the view to improve mental health outcomes of mothers and offspring during the postnatal period and beyond.
